# Etiology- and age-specific timing of death by neurologic criteria evaluation and declaration in clinical practice

**DOI:** 10.1186/s13054-026-06069-8

**Published:** 2026-05-09

**Authors:** Kristian Barlinn, Daniela Schoene, Konrad Pleul, Martin Roessler, Andre Worm, Norma J. Diel, Patrick Schramm, Felix Pfeifer, Hagen B. Huttner, Axel Rahmel

**Affiliations:** 1 Department of Neurology, Medical Faculty and University Hospital Carl Gustav Carus, TUD Dresden University of Technology, Fetscherstraße 74, 01307 Dresden, Germany; 2 German Organ Procurement Organization, Frankfurt am Main, Germany; 3 BARMER Institute for Health Care System Research (bifg), Berlin, Germany

**Keywords:** Death by neurologic criteria, Brain death, Etiology, Age, Time to event, Brain injury, Organ donation

## Abstract

**Background:**

The timing of declaration of death by neurologic criteria (DNC) after acute brain injury varies in clinical practice. However, large-scale data describing etiology- and age-specific timing of DNC evaluation and declaration are limited. This study aimed to characterize these timing patterns in a cohort of patients with DNC.

**Methods:**

Retrospective cohort study using registry data from German hospitals reporting cases of guideline-confirmed DNC to the national organ procurement organization. Included were adults (≥ 18 years) who were declared DNC between April 1, 2020, and August 31, 2025. Brain injury was categorized by ICD-10 as traumatic brain injury (TBI), intracerebral hemorrhage (ICH), subarachnoid hemorrhage (SAH), acute ischemic stroke (AIS), hypoxic-ischemic encephalopathy (HIE). The primary outcome was time from initiation of mechanical ventilation to DNC declaration; time to first clinical examination fulfilling criteria for DNC was analyzed as a secondary outcome. Time-to-event distributions were summarized using cumulative incidence estimates, and associations of timing with etiology, age, and selected clinical variables were explored using random survival forests.

**Results:**

Among 6,701 patients with DNC from 716 hospitals, 6,397 had complete timing data (median age, 59 years [IQR, 46–69]; 54% male). Median time to DNC differed across etiologies (*p* < 0.001): 55.5 h for ICH, 58.4 h for TBI, 65.9 h for SAH, 89.9 h for HIE and 96.8 h for AIS. By 72 h of ventilation, cumulative incidence of DNC was 64.5% (95%CI, 61.3–67.5) in TBI, 62.4% (95%CI, 59.9–64.7) in ICH, 53.7% (95%CI, 50.9–56.4) in SAH, 36.7% (95%CI, 34.4–38.9) in HIE and 31.9% (95%CI, 28.3–35.4) in AIS. By 120 h, cumulative incidence reached 83.9% (95%CI, 81.3–86.1) in TBI, 81.5% (95%CI, 79.5–83.4) in ICH, 69.0% (95%CI, 66.4–71.5) in SAH, 67.3% (95%CI, 65.0-69.4) in HIE and 65.7% (95%CI, 61.9–69.1) in AIS. Older age was associated with shorter time to DNC in TBI and ICH, whereas associations were minimal in AIS and HIE.

**Conclusions:**

The timing of DNC evaluation and declaration varies substantially across etiologies and age groups. These findings may help to better understand temporal patterns of DNC determination among patients with DNC in clinical practice.

**Supplementary Information:**

The online version contains supplementary material available at 10.1186/s13054-026-06069-8.

## Background

Death by neurologic criteria (DNC) is defined as the irreversible cessation of all brain function, including the brainstem [1]. It is a medical and legal criterion for declaration of death in many countries and represents a critical threshold in the management of patients with devastating brain injury [2]. Death by neurologic criteria most commonly results from acute neurological events such as traumatic brain injury (TBI), intracerebral hemorrhage (ICH), subarachnoid hemorrhage (SAH), acute ischemic stroke (AIS) or hypoxic-ischemic encephalopathy (HIE) [3]. 

Although diagnostic protocols for DNC are clearly defined, the timing of DNC evaluation and formal declaration remains variable in clinical practice. Progression from severe brain injury to DNC may take several days and potential differences in the temporal course of DNC determination across etiologies are not well characterized. Current guidelines emphasize ongoing monitoring and repeated evaluations but do not consistently define minimum observation periods [4, 5]. Some recommendations propose a minimum of 72 h in selected cases - such as after HIE - but these are largely based on expert opinion and are not uniformly applied [6]. Prediction tools from post-cardiac arrest care show limited generalizability [7, 8]. This lack of standardization may contribute to variability in the timing of DNC evaluation and formal declaration in clinical practice.

In this context, understanding real-world patterns of DNC determination in clinical practice is important. Although diagnostic pathways are well defined, the timing of DNC evaluation and formal declaration may vary across clinical settings. However, large-scale data describing these temporal patterns in routine clinical practice are limited. We therefore aimed to characterize etiology- and age-specific timing of DNC evaluation and formal declaration in a cohort of patients with DNC.

## Methods

### Study design and data source

We conducted a retrospective cohort study using prospectively collected registry data from the German national organ procurement organization (OPO). Adults (≥ 18 years) with DNC between April 1, 2020, and August 31, 2025, were included. All patients had received mechanical ventilation prior to death and were diagnosed with DNC according to the German Medical Association guideline [5]. Hospitals are mandated by law to notify the national OPO of cases in which DNC has been confirmed and organ donation is considered a possibility. Upon notification, the OPO prospectively reviews each case for organ donation eligibility and documents clinical and procedural information in a standardized registry, which served as the data source for this analysis. DNC determinations were performed and documented by the treating physicians in accordance with the German Medical Association guideline [5]. For the purposes of this study, DNC diagnoses were not independently re-adjudicated or centrally verified beyond the established clinical and regulatory framework. Data collection within the registry was performed by trained OPO coordinators who are responsible for case evaluation and documentation in the organ donation process. These coordinators are healthcare professionals, typically physicians or specialized nurses, who have completed additional training in organ donation procedures, including certification through structured programs coordinated by regional physician regulatory bodies. Hospitals included in this study represent centers that reported at least one case of DNC to the national OPO during the study period. In Germany, approximately 1,200 hospitals are legally required to notify the OPO of such cases according to the German Transplantation Act. Thus, the analyzed cohort reflects reporting centers with at least one eligible case during the study period.

This study was approved by the Ethics Committee of the Technical University Dresden (EK-373082025); the requirement for informed consent was waived because the analysis was based on anonymized secondary data. The Strengthening the Reporting of Observational Studies in Epidemiology (STROBE) reporting guidelines were followed [9]. 

### DNC determination framework

According to the German Medical Association guideline, determination of DNC requires fulfillment of predefined prerequisites, including evidence of a severe and irreversible brain injury, exclusion of confounding factors such as sedation, intoxication, metabolic disturbances or hypothermia [5]. Clinical examination includes demonstration of coma, absence of brainstem reflexes and apnea testing to confirm loss of respiratory drive. In cases of primary supratentorial lesions or secondary brain injuries (i.e., non-primary structural brain injury, including hypoxic-ischemic brain injury following cardiac arrest or other forms of diffuse global brain injury), irreversibility must be confirmed by a repeat clinical examination after a defined observation period (≥ 12 h for primary supratentorial injury and ≥ 72 h for secondary injury). In cases of primary infratentorial lesions or when clinical examination cannot be reliably completed, ancillary testing is mandatory. Although guideline-based indications for ancillary testing are limited to these situations, its use is also permitted in other contexts and is frequently applied in routine clinical practice. In such cases, ancillary testing may replace repeat clinical examination and thereby obviate the observation period. There is no fixed interval between clinical examination and ancillary testing once prerequisites are fulfilled. Approved ancillary methods include electroencephalography, evoked potentials, Doppler ultrasonography, perfusion scintigraphy and computed tomography angiography [5]. 

### Variables and definitions

For all included cases, data were available on patient demographics (age and sex), admission date, initiation of mechanical ventilation, times of clinical examinations and ancillary testing, time of DNC declaration and ICD-10 diagnoses judged by the treating physicians as causally related to DNC. These codes were mapped for this analysis to major etiologic categories, including spontaneous ICH, AIS, SAH, HIE, TBI, encephalitis/meningitis, cerebral venous thrombosis and tumor. Cases that could not be clearly assigned to any category were classified as unknown due to limitations in registry-based categorization rather than the absence of a clinically identified cause at the time of DNC declaration. Additionally, the dataset included information on the occurrence of deceased organ donation. Implausible data were defined as inconsistent or non-physiological time intervals in recorded timestamps used for time-to-event analyses (e.g., DNC declaration preceding initiation of mechanical ventilation). The primary analysis cohort was defined based on the availability of valid data for the calculation of time from initiation of mechanical ventilation to DNC declaration. Patients with missing or implausible data for this time interval were excluded from the primary analysis. For the secondary outcome (time to first clinical examination), analyses were restricted to patients with available data for this variable.

### Outcomes

The primary outcome was the time interval, measured in hours, from the initiation of mechanical ventilation to the formal declaration of DNC, hereafter referred to as time-to-DNC interval. This interval captures the clinically relevant period beginning with the initiation of ventilatory support - typically necessitated by severely impaired consciousness or respiratory failure - and ending with the diagnosis of DNC. Cases lacking the required timestamps were excluded a priori. The secondary outcome was the time from initiation of mechanical ventilation to the first clinical examination fulfilling criteria for DNC. For the purposes of this study, this refers to the first clinical examination consistent with DNC as documented in the registry. This measure was included to enable international comparability, as some jurisdictions (e.g., the United States) allow DNC determination based on a single clinical examination [10]. In such settings, time to DNC in our dataset corresponds to time-to-first-examination.

### Statistical analysis

Descriptive analyses were performed by summarizing categorical variables as frequencies and continuous variables as mean ± standard deviation or median (interquartile range [IQR]), depending on data distribution. Time-to-event distributions were visualized using log-transformed boxplots by etiology and cumulative incidence curves with 95% confidence intervals (95% CIs), based on the asymptotic normal distribution of the log-transformed estimates. Comparisons of time metrics between multiple groups (e.g., etiologies) were performed using the Kruskal-Wallis test, whereas comparisons between two groups (e.g., organ donors vs. non-donors) were performed using the Wilcoxon rank-sum test. Due to small sample sizes and heterogeneity, rare etiologies were not included in inferential statistical comparisons but were retained for descriptive analyses and are presented in supplementary cumulative incidence curves.

The primary analysis focused on time from initiation of mechanical ventilation to DNC declaration. The secondary analysis examined time to first clinical examination fulfilling criteria for DNC, using analogous methods. In addition, the interval between completion of the first clinical examination fulfilling criteria for DNC and formal DNC declaration was calculated.

Exploratory analyses were conducted using random survival forests (RSF), with separate models fitted for each outcome (time-to-DNC and time to first clinical examination) [11]. RSF are nonparametric, tree-based models that allow flexible modeling of time-to-event data without assuming proportional hazards. Models were fitted using 500 trees, a node size of 10 and a maximum of 20 random splits per predictor. The following variables were included as predictors: type of brain injury, age, sex, university hospital status and calendar year of ventilation; for the time-to-DNC model, ancillary testing was included as an additional predictor. These variables were selected based on data availability and presumed clinical relevance. Variable importance was assessed using permutation-based measures. To visualize predictive relationships between DNC, type of brain injury and age, predicted cumulative incidence curves were derived from the fitted RSF model, assuming a female patient treated in a university hospital in 2025. Model performance was assessed using Harrell´s concordance index (C-index) [12]. These analyses were intended to explore relative variable importance rather than to develop a predictive model. Finally, as an exploratory descriptive analysis, between-hospital variability in timing was assessed. Hospital-level heterogeneity in median time-to-DNC and time-to-first clinical examination was visualized using density plots. In addition, log-linear multilevel models with a normally distributed random intercept at the hospital level were applied. No additional covariates were included, as the aim was to descriptively quantify variability rather than to assess adjusted associations. Intracluster correlation coefficients (ICCs) were derived to estimate the proportion of variance attributable to the hospital level. Subgroup analyses comparing organ donors and non-donors were performed for time-to-event outcomes. All statistical tests were two-sided and p-values < 0.05 were considered statistically significant. Analyses were performed using R (version 4.3.2).

## Results

### Study cohort

Over the five-year study period, 6,701 patients with DNC were reported to the national OPO from 716 hospitals across Germany. Of these, 304 patients were excluded because of missing or implausible data on the timing of mechanical ventilation initiation, leaving 6,397 patients for the primary analysis of time to DNC declaration. The median age was 59 years (IQR, 46–69) and 54% were male. The majority of DNC cases were attributed to HIE (27.8%), spontaneous ICH (24.2%), SAH (19.9%), TBI (14.4%) and AIS (10.4%). Table 1 summarizes the baseline characteristics of the study cohort. Comparisons of baseline characteristics between patients with complete and missing timing data are presented in eTable 1.

**Table 1:** Study cohort characteristics

Variable	Total (*n* = 6,397)
Age, median (IQR), years	59 (46–69)
Age range, years	18–96
Sex, n (%)	
Male	3,472 (54.3)
Female	2,925 (45.7)
Hospital type, n (%)	
University hospital	2,157 (33.7)
Non-university hospital	4,240 (66.3)
Etiology of brain injury, n (%)	
Hypoxic-ischemic encephalopathy	1,776 (27.8)
Intracerebral hemorrhage	1,550 (24.2)
Subarachnoid hemorrhage	1,275 (19.9)
Traumatic brain injury	919 (14.4)
Acute ischemic stroke	667 (10.4)
Meningitis/encephalitis	73 (1.1)
Brain tumor	48 (0.8)
Subdural hematoma	35 (0.6)
Cerebral venous thrombosis	13 (0.2)
Unknown	41 (0.6)
Mode of DNC confirmation, n (%)^*^	
By second clinical examination	901 (14.2)
By ancillary testing	5,422 (85.8)
CT-angiography	2,316 (36.6)
Electroencephalography	2,126 (33.6)
Transcranial Doppler / Duplex	754 (11.9)
Cerebral perfusion scintigraphy	179 (2.8)
Somatosensory / auditory evoked potentials	48 (0.8)
Donor after DNC, n (%)	4,677 (73.1)

IQR=interquartile range; DNC=death by neurologic criteria. ^*^Percentages are based on patients with available data on the mode of DNC confirmation (*n* = 6,323)

### Time to declaration of DNC

**Fig. 1:**
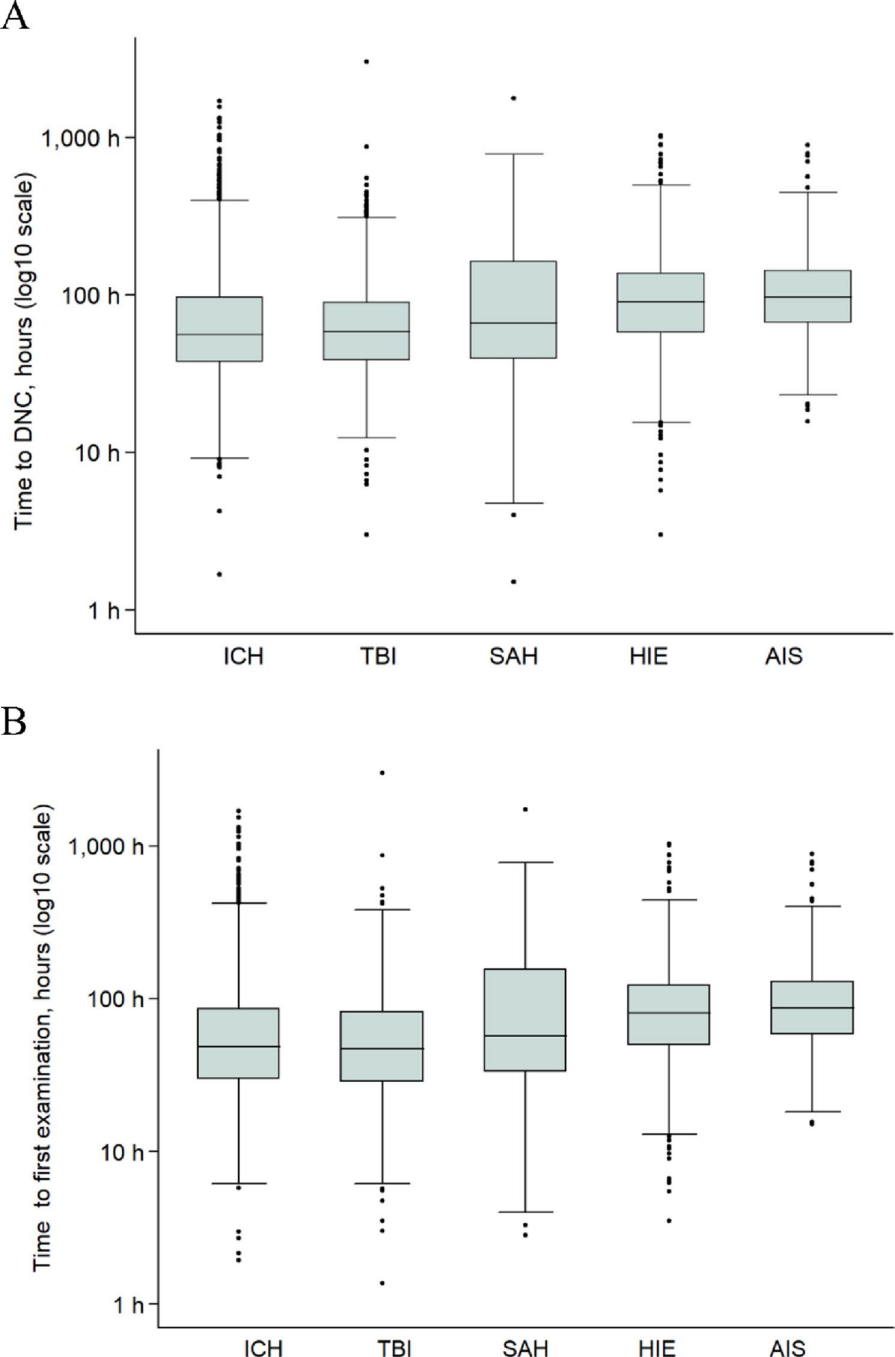
Time to event distributions by etiology. (**A**) Boxplots of log10-transformed time (hours) from initiation of mechanical ventilation to (**A**) declaration of death by neurologic criteria (DNC) and (**B**) clinical examination (“single exam equivalent”), stratified by etiology. Boxes show IQRs on the log10 scale with medians; whiskers extend to 1.5×IQR

**Fig. 2:**
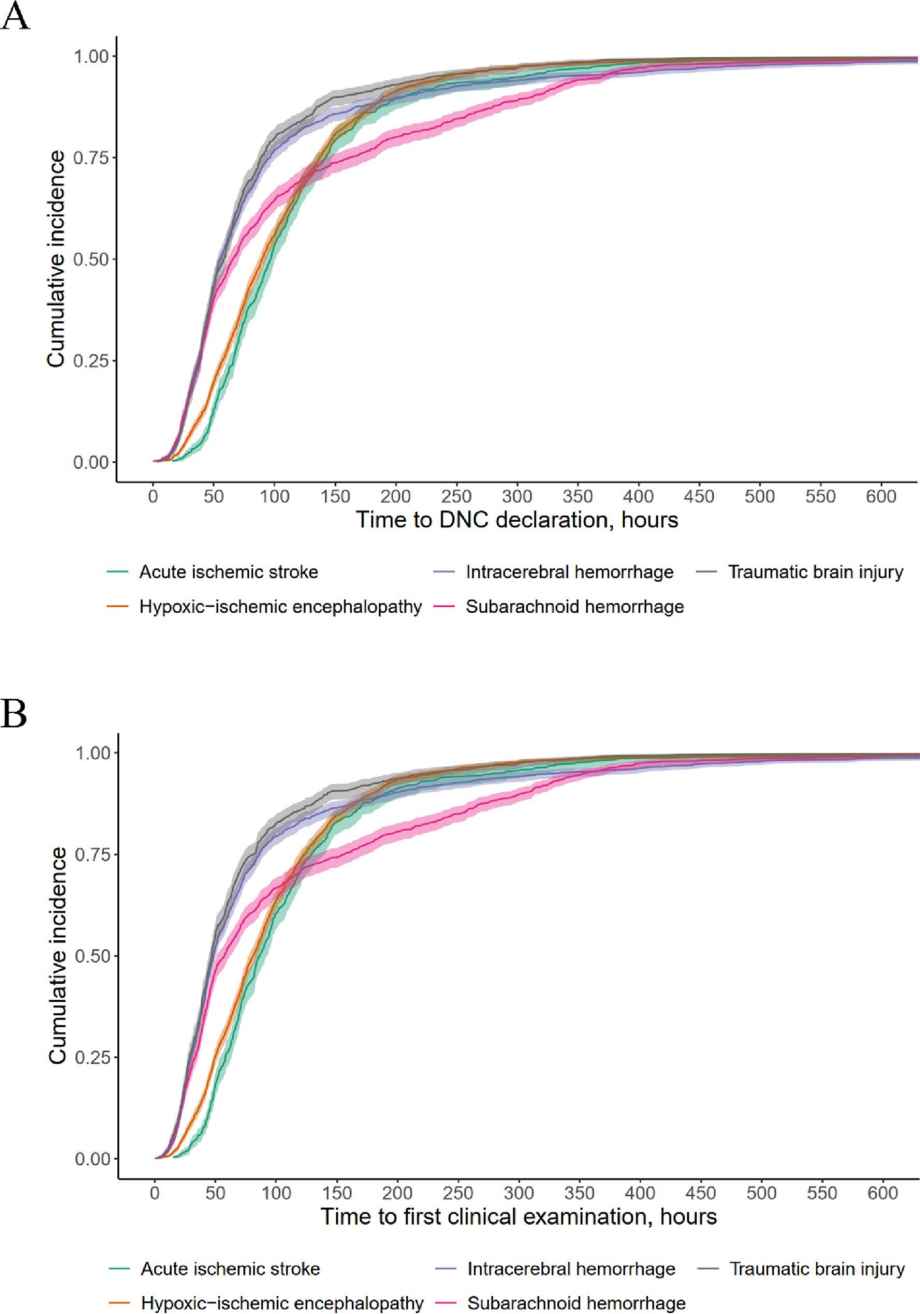
Cumulative incidence of DNC declaration and first clinical examination by etiology. (**A**) Time from initiation of mechanical ventilation to DNC declaration. (**B**) Time from initiation of mechanical ventilation to clinical examination (“single-exam equivalent”). Shaded bands represent 95% confidence intervals. Curves differ by etiology, showing the fastest progression in traumatic and hemorrhagic brain injury and the slowest in hypoxic–ischemic and ischemic etiologies

The overall median time from initiation of mechanical ventilation to DNC declaration was 71.9 h (IQR, 43.8–125.0). Median time-to-DNC varied across etiologies (*p* < 0.001), with medians (IQR) of 55.5 h (37.3–96.5) for ICH, 58.4 h (38.4–89.1) for TBI, 65.9 h (39.1-162.2) for SAH, 89.9 h (57.2-137.2) for HIE and 96.8 h (66.3-142.7) for AIS. Figure 1A shows log-scale boxplots stratified by injury type. By 72 h of ventilation, the cumulative incidence of DNC was 64.5% (95%CI, 61.3–67.5) in TBI, 62.4% (95%CI, 59.9–64.7) in ICH, 53.7% (95%CI, 50.9–56.4) in SAH, 36.7% (95%CI, 34.4–38.9) in HIE, and 31.9% (95%CI, 28.3–35.4) in AIS. By 120 h of ventilation, the cumulative incidence was 83.9% (95%CI, 81.3–86.1) in TBI, 81.5% (95%CI, 79.5–83.4) in ICH, 69.0% (95%CI, 66.4–71.5) in SAH, 67.3% (95%CI, 65.0-69.4) in HIE and 65.7% (95%CI, 61.9–69.1) in AIS. Cumulative incidence curves are shown in Fig. 2A. Rare etiologies comprised 2.7% of cases overall; supplementary cumulative incidence curves for all etiologies are shown in the Supplement (eFigure 1). Deceased organ donation occurred in 4,677 (73.1%) of 6,397 cases, with no significant differences in time-to-DNC between donors and non-donors, both overall and within each etiology subgroup (all *p* > 0.05).

### Time to first clinical examination for determination of DNC

Complete data on time to first clinical examination were available for 6,307 patients. The overall median was 63.9 h (IQR, 37.8-114.1). Times differed by etiology, with medians (IQR) of 47.0 h (28.7–82.7) for TBI, 48.3 h (29.8–86.0) for ICH, 57.0 h (33.4-155.5) for SAH, 81.0 h (49.8–123.0) for HIE and 86.6 h (58.2-128.9) for AIS (*p* < 0.001). Log-scale boxplots for the first examination are shown in Fig. 2B. Within the first 72 h of ventilation, the cumulative incidence of a completed first examination was 72.1% (95%CI, 69.0-74.8) in TBI, 68.1% (95%CI, 65.7–70.4) in ICH, 58.0% (95%CI, 55.2–60.7) in SAH, 42.8% (95%CI, 40.4–45.1) in HIE, and 39.3% (95%CI, 35.5–42.9) in AIS. By 120 h of ventilation, the cumulative incidence was 86.0% (95%CI, 83.5–88.1) in TBI, 83.2% (95%CI, 81.2–84.9) in ICH, 70.7% (95%CI, 68.1–73.2) in SAH, 73.9% (95%CI, 71.7–75.9) in HIE and 70.9% (95%CI, 67.2–74.1) in AIS. Cumulative incidence curves are shown in Fig. 2B. Time-to-first-examination did not differ between donors and non-donors (all *p* > 0.05). The median interval between completion of the first clinical examination and the final DNC declaration was 2.4 h (IQR, 1.2–6.5).

### Predictor importance and random-survival-forest analysis

**Fig. 3:**
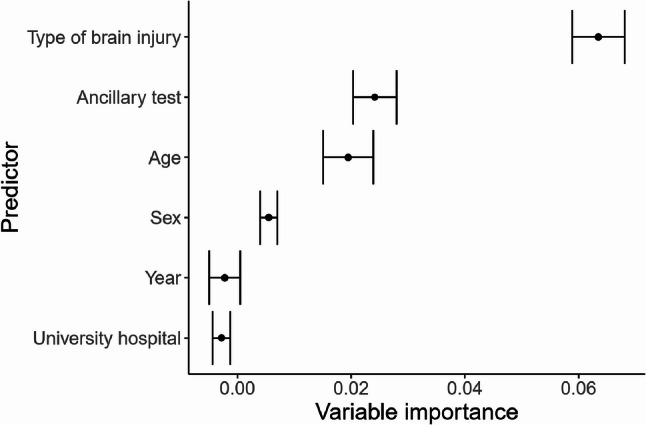
Variable importance for predictors of time to DNC determination. Permutation-based variable importance from the random survival forest model. Type of brain injury showed the highest permutation importance for time from initiation of mechanical ventilation to DNC declaration, followed by ancillary testing and age. Sex, university hospital status, and calendar year showed lower importance, with confidence intervals overlapping zero. Dots represent mean importance estimates and horizontal bars indicate 95% confidence intervals

**Fig. 4:**
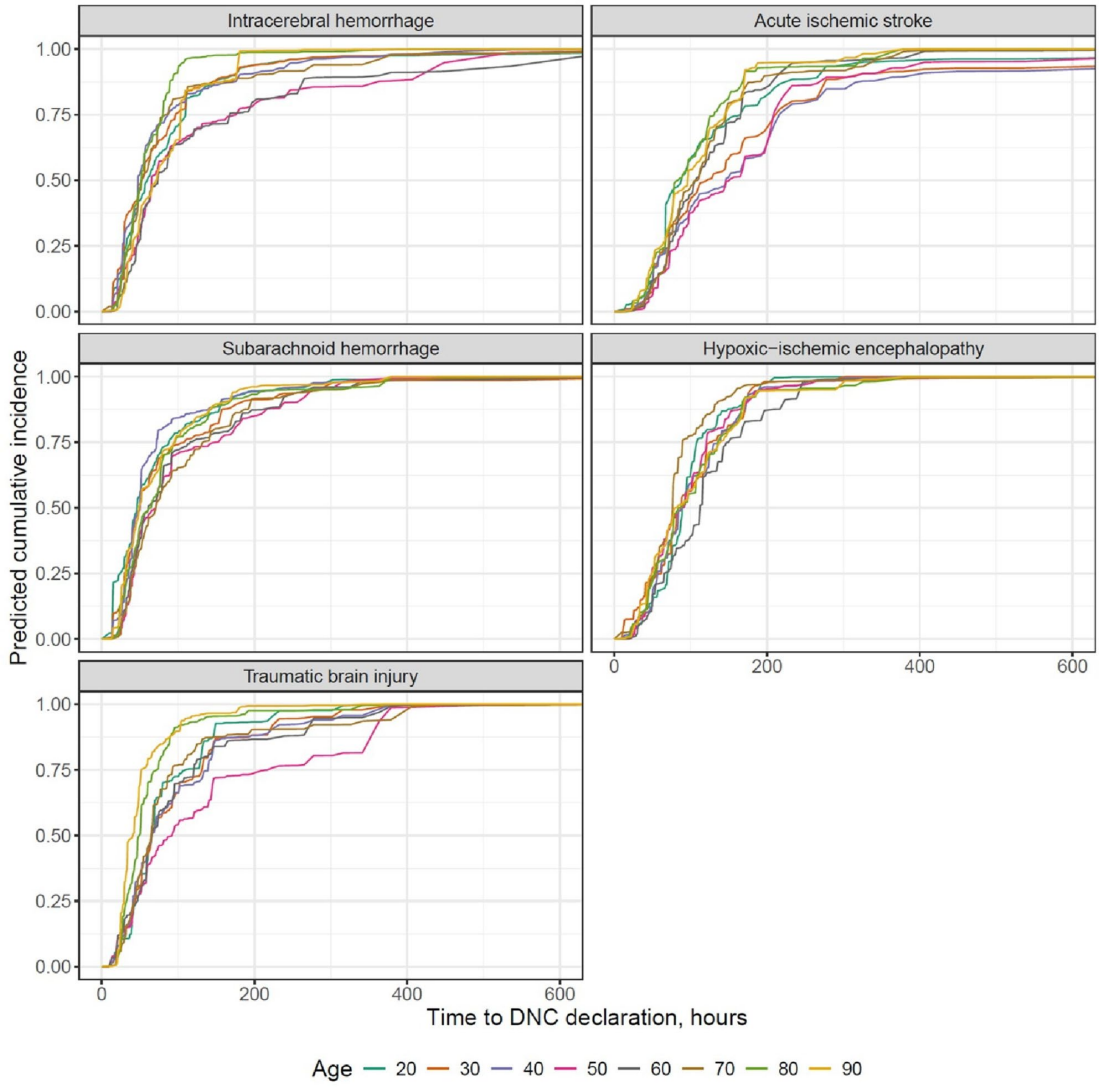
Predicted cumulative incidence of DNC declaration by age and etiology. Predicted cumulative incidence curves from the random survival forest model showing the time from initiation of mechanical ventilation to DNC declaration across age groups and brain injury etiologies. Predictions assume a female patient ventilated in a university hospital in 2025

In the RSF analysis, type of brain injury showed the highest permutation importance for time-to-DNC, followed by ancillary testing and age, whereas sex, university hospital status and calendar year showed lower permutation importance with confidence intervals overlapping zero (Fig. 3). Predicted cumulative incidence curves by age and etiology are shown in Fig. 4. In TBI and ICH, curves for older patients were located at earlier time points compared to younger patients, whereas in AIS and HIE, curves showed minimal separation across age groups. For SAH, curves showed modest separation between age strata. The C-index was 0.44 for both time-to-first-examination and time-to-DNC.

### Hospital-level heterogeneity

Hospital-specific median times-to-DNC and times-to-first-examination are shown in eFigures 2 and 3. The ICCs derived from the estimated multilevel models indicated that approximately 7% of the total variance of both the log-transformed times-to-first-examination and times-to-DNC was attributable to the hospital level.

## Discussion

This large cohort of almost 6,400 patients with DNC provides detailed insights into etiology- and age-specific patterns of DNC determination. We observed substantial variability in the time from initiation of mechanical ventilation to DNC declaration across etiologies, with shorter intervals in traumatic and hemorrhagic injuries and longer intervals in hypoxic-ischemic and ischemic etiologies. Age further modified these patterns, particularly in traumatic and hemorrhagic injuries, whereas age showed limited association with timing in ischemic or hypoxic-ischemic etiologies. Donor status was not associated with DNC timing.

From a biological perspective, primary hemorrhagic or traumatic injuries are typically characterized by abrupt intracranial pressure surges and rapid herniation, resulting in early circulatory arrest of the brain, which may be associated with a shorter time intervals to DNC declaration [13]. In contrast, hypoxic-ischemic injury often leads to delayed, progressive cytotoxic edema and uncertain prognostic evolution, prompting prolonged observation [14]. The longer time intervals observed after SAH may reflect a more variable clinical course, with initial stabilization in some patients followed by delayed secondary brain injury such as vasospasm or delayed cerebral ischemia [15]. However, as DNC determination also requires legal and procedural confirmation, the observed intervals do not necessarily reflect the exact physiological moment of irreversible brain failure but rather diagnostic and procedural timelines shaped by resource availability, clinician expertise and ancillary testing practices [16]. These findings highlight variability in the timing of DNC evaluation and declaration in routine clinical practice. Among patients who developed DNC following TBI or ICH, 60% were declared deceased within 72 h of initiation of mechanical ventilation, compared to 40% in patients who developed DNC following HIE or AIS. Importantly, these observations are restricted to patients with confirmed DNC and do not capture the broader population of patients with acute brain injury who do not progress to DNC.

The distribution of brain injuries leading to DNC in this cohort aligns with previous reports, with hypoxic-ischemic and hemorrhagic lesions representing the predominant etiologies [3]. Rare etiologies such as cerebral venous thrombosis or central nervous system infections accounted for only a small proportion of cases, likely reflecting both their lower incidence and their typically more heterogeneous and often more favorable clinical course. Published data on temporal dynamics of DNC remain limited. In a Korean cohort of 414 patients with DNC, the mean interval from injury onset to DNC declaration was 8.5 ± 7.7 days, with slower progression observed in patients exhibiting spontaneous movements or those who had recently undergone craniotomy [17]. Similarly, a Turkish single-center series (*n* = 44) reported a mean interval of 7.9 days from ICU admission to DNC declaration [18]. In a recent U.S. study of 569 patients, the mean time from neurological event to DNC declaration ranged from 55 to 76 h depending on whether single- or double-examination protocols were used [10]. Importantly, these studies did not assess etiology-specific trajectories and often used symptom onset or ICU admission as time zero - both of which are prone to variability due to uncertain timing and differing clinical pathways. In contrast, we defined time from initiation of ventilation, a clinically relevant reference point aligned with donor identification workflows [19]. 

Building on this methodological approach, we further explored procedural factors influencing DNC timing within the German framework. The short interval between the first clinical examination and DNC declaration (median 2.4 h) likely reflects the predominant use of ancillary testing in Germany, applied in 86% of cases, enabling immediate confirmation once clinical prerequisites are met [5, 19]. While ancillary testing is mandated in specific scenarios, it is also frequently used to confirm irreversibility without completing the full observation period. The timing of the first examination (“single-exam equivalent”) closely paralleled formal DNC confirmation, with nearly identical cumulative incidence patterns across etiologies. This supports the notion that procedural factors, such as repeat examinations and ancillary testing logistics, contribute to the interval between clinical assessment and formal DNC declaration. Comparable findings have been reported in a U.S. cohort, where adoption of a single-examination protocol reduced time to DNC declaration by 21 h without affecting diagnostic accuracy or donor yield [10]. In our cohort, approximately one quarter of patients with DNC did not proceed to organ donation, likely reflecting consent regulations in Germany and medical suitability.

Age-related differences in the timing of DNC evaluation and declaration were heterogeneous across etiologies and likely reflect both biological and treatment-related factors. In TBI and ICH, higher age was associated with shorter time to DNC, whereas in ischemic and hypoxic-ischemic etiologies, age showed little or no association with timing. These heterogeneous relationships suggest that age-related influences on DNC determination within patients with DNC are not uniform but depend on injury type. Shorter times observed in older individuals may reflect differences in treatment goals and care intensity rather than purely biological acceleration of cerebral injury. In older patients, devastating hemorrhagic or traumatic injuries may more often lead to earlier treatment limitation or less aggressive surgical intervention, whereas younger patients more frequently undergo prolonged neurocritical care, potentially delaying DNC evaluation and declaration.

This study has several important strengths. To our knowledge, it represents the largest cohort to date, comprising nearly 6,400 patients with DNC across all major etiologies of acute brain injury. The use of standardized registry data ensures high validity of documented time points and representativeness across different hospital types and regions. By analyzing both the time to formal DNC declaration and the time to first clinical examination, this study enables international comparability with countries applying single-examination protocols [10]. However, several limitations should be acknowledged. First, this was a secondary analysis of registry data, which may vary in quality and documentation across hospitals. In addition, as the registry includes only cases reported to the organ procurement organization and notification in the German system is closely linked to the consideration of organ donation, cases in which organ donation was not considered may be underrepresented, introducing potential selection bias. DNC determinations were performed as part of routine clinical practice and were not independently re-adjudicated, potentially introducing variability related to differences in clinical assessment and documentation [20]. Second, the timing of DNC evaluation reflects real-world clinical practice and does not necessarily correspond to the earliest possible time at which DNC criteria may have been fulfilled. Rather, it captures the timing of evaluation and formal declaration as determined by clinicians in routine care. The initiation of DNC evaluation may be influenced by clinical, organizational and contextual factors, including clinician availability, timing of assessment and other practical considerations that are not captured in the registry. Third, participating hospitals likely differed in neurocritical care expertise, access to ancillary testing and procedural workflows, which may have influenced timing. Fourth, important clinical confounders such as sedation, metabolic disturbances or prior therapeutic interventions were not available in the registry. Fifth, classification of brain injury types relied on ICD-10 coding and may not fully capture clinical complexity. Sixth, data for the secondary outcome were incomplete and may not be missing at random; findings related to this outcome should therefore be interpreted with caution. Seventh, data availability on potential predictors for time-to-event analyses was limited. As a result, important variables could not be included in the RSF models, which showed limited predictive performance; variable importance measures should therefore be interpreted cautiously. Finally, this analysis included only patients with DNC. The findings cannot be extrapolated to patients with acute brain injury at risk of progressing to DNC and should be interpreted as descriptive and hypothesis-generating.

## Conclusions

This study provides a comprehensive characterization of etiology- and age-specific timing of DNC evaluation and declaration in routine clinical practice. These findings may help to better understand the timing of DNC evaluation and declaration in clinical practice but should not be interpreted as predictive for individual patients or extrapolated to broader populations at risk of DNC.

## Supplementary Information

Below is the link to the electronic supplementary material.

Supplementary Material 1.

Supplementary Material 2.

## Data Availability

Anonymized data that are not published within this article may be made available to qualified researchers upon reasonable request to the corresponding author, subject to approval by the national OPO and participating hospitals.

## Abbreviations

AIS: Acute ischemic stroke
DNC: Death by neurologic criteria
HIE: Hypoxic-ischemic encephalopathy
ICH: Intracerebral hemorrhage
IQR: Interquartile range
OPO: Organ procurement organization
RSF: Random survival forests
SAH: Subarachnoid hemorrhage
STROBE: Strengthening the reporting of observational studies in epidemiology
TBI: Traumatic brain injury
