# Supplementary material for: Etiology- and age-specific timing of death by neurologic criteria evaluation and declaration in clinical practice
Source: Crit Care. 2026 May 9;30:246. doi: 10.1186/s13054-026-06069-8 (PMC13159350; doi:10.1186/s13054-026-06069-8)
Supplement: Supplementary file 2 — Supplementary Material 2. [file 13054_2026_6069_MOESM2_ESM.docx]

**Supplement**

**eTable 1.** Baseline characteristics of the main analysis population (time to DNC) and patients with missing timing data

| Variable | Complete timing data (n=6,397) | Missing timing data (n=301) |
| --- | --- | --- |
| Age, median (IQR), years | 59 (46–69) | 58 (45-69) |
| Sex, male. n (%) | 3,472 (54.3) | 168 (55.3) |
| Etiology of brain injury, n (%) |  |  |
| Hypoxic-ischemic encephalopathy | 1,776 (27.8) | 90 (29.6) |
| Intracerebral hemorrhage | 1,550 (24.2) | 78 (25.7) |
| Subarachnoid hemorrhage | 1,275 (19.9) | 59 (19.4) |
| Traumatic brain injury | 919 (14.4) | 34 (11.2) |
| Acute ischemic stroke | 667 (10.4) | 23 (7.6) |
| Meningitis/encephalitis | 73 (1.1) | 3 (1) |
| Brain tumor | 48 (0.8) | 1 (0.3) |
| Subdural hematoma | 35 (0.6) | 6 (2) |
| Cerebral venous thrombosis | 13 (0.2) | 1 (0.3) |
| Unknown | 41 (0.6) | 8 (2.9) |

IQR=interquartile range

**eFigure 1.** Cumulative incidence curves for DNC declaration in all etiologic categories


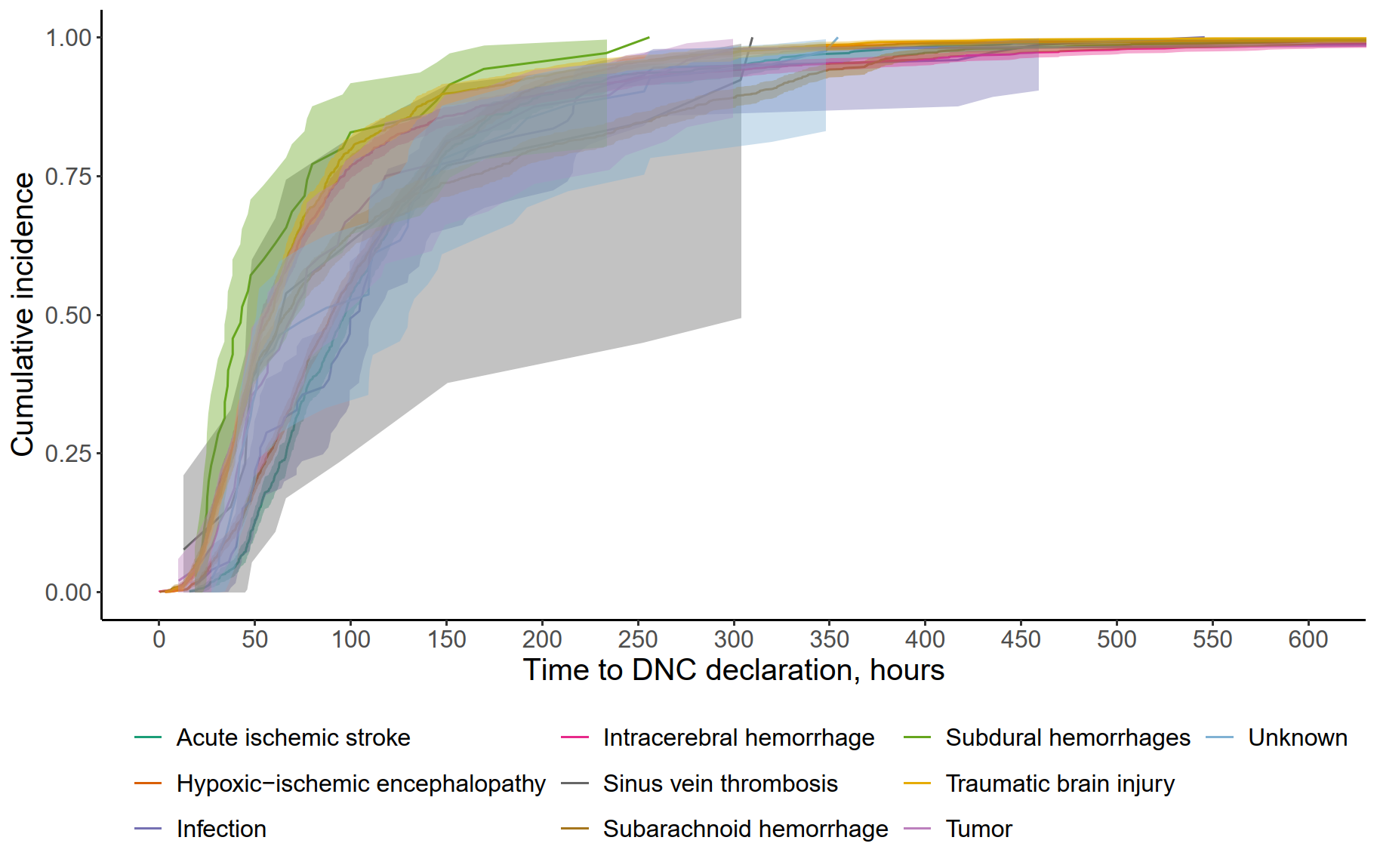


**eFigure 1.** Proportion reaching DNC from initiation of mechanical ventilation, shown for all categories. Etiologies are highlighted with 95% confidence bands; Axes: x-axis, time (h); y-axis, proportion reaching DNC.

**eFigure 2.** Hospital-level variation in median time to DNC declaration by etiology


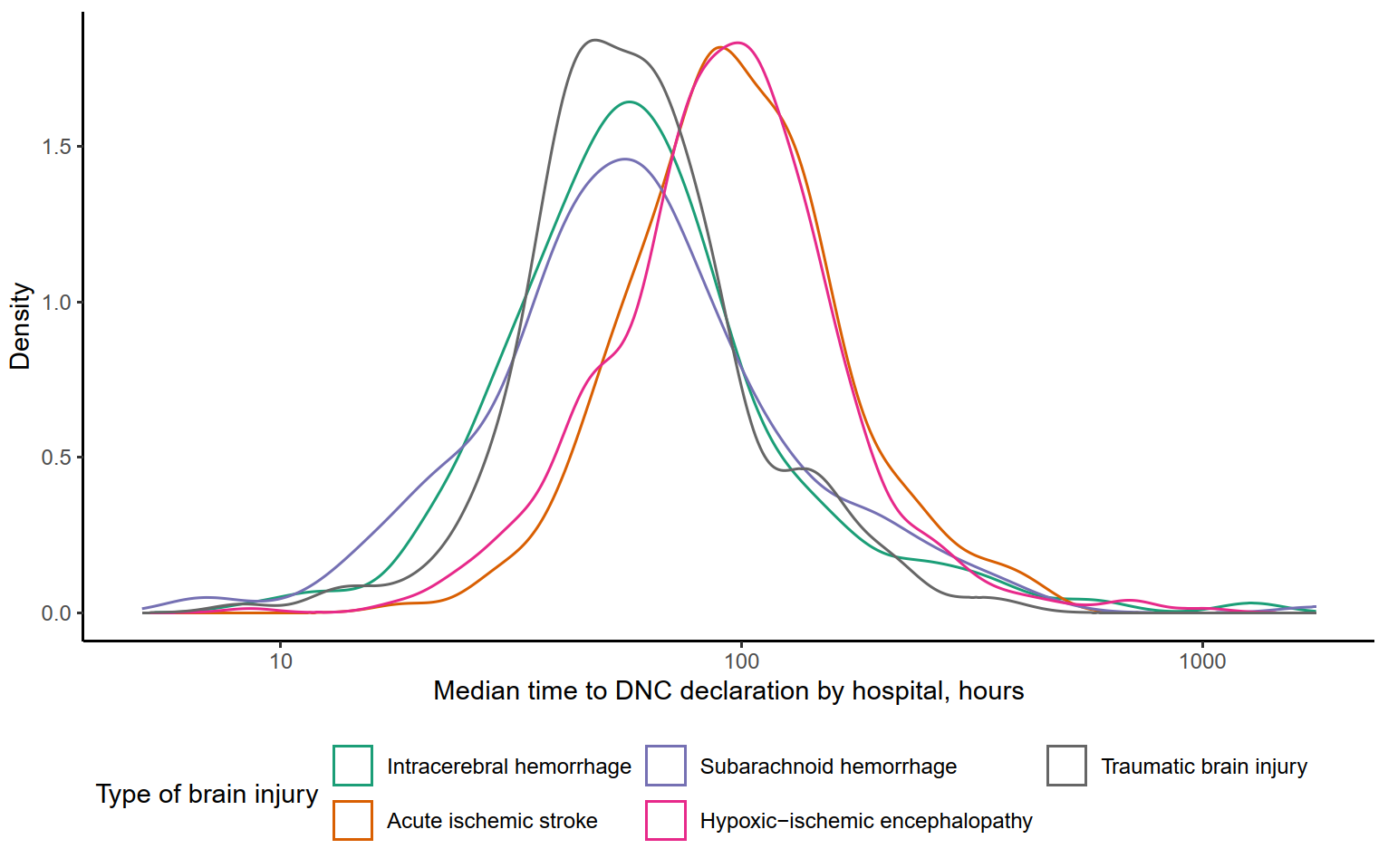


**eFigure 2.** Density plots showing the distribution of hospital-specific median time from initiation of mechanical ventilation to DNC declaration, stratified by type of brain injury. Each curve represents the distribution of median times across hospitals for a given etiology.

**eFigure 3.** Hospital-level variation in median time to first clinical examination by etiology


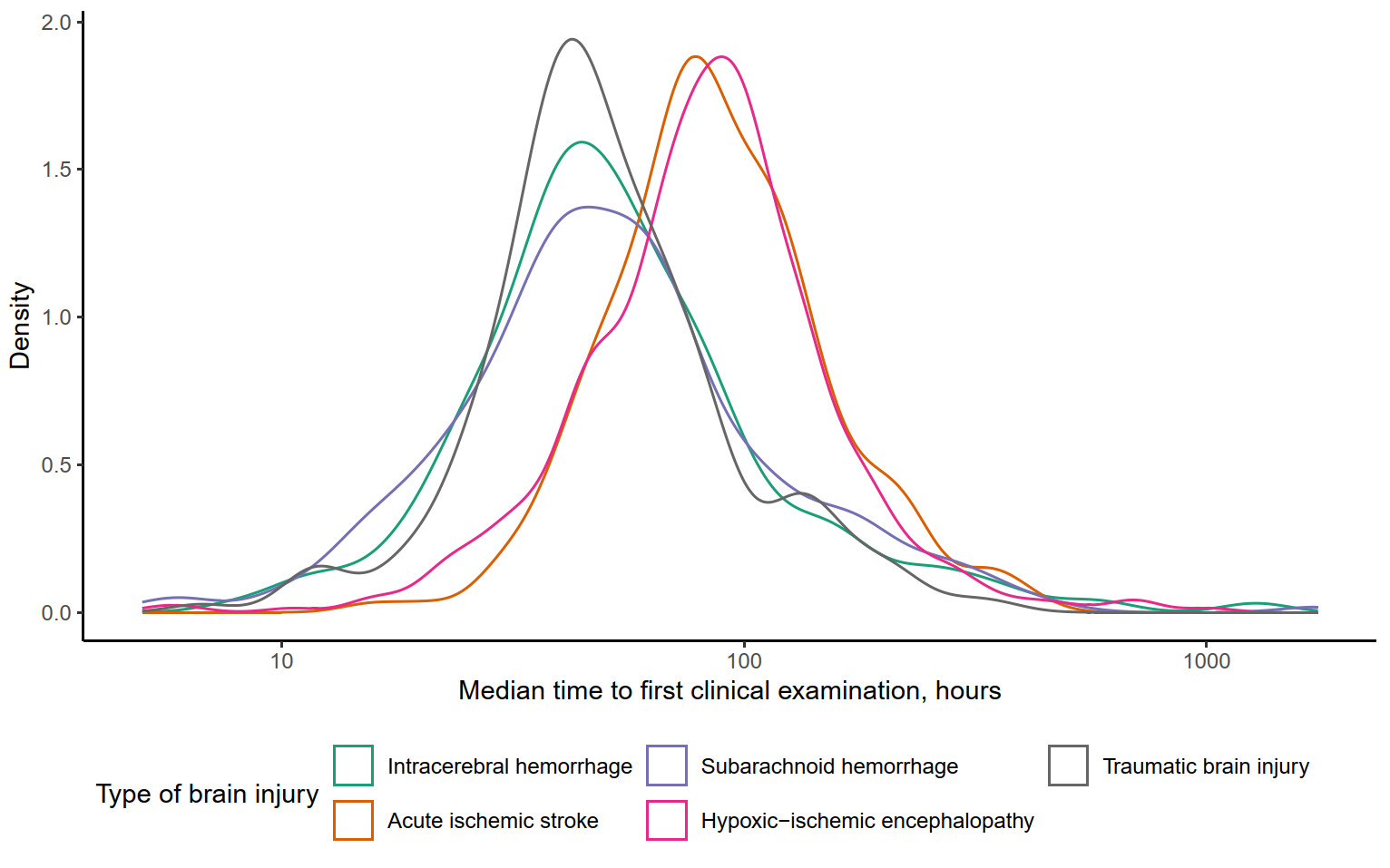


**eFigure 3.** Density plots showing the distribution of hospital-specific median time from initiation of mechanical ventilation to first clinical examination, stratified by type of brain injury. Each curve represents the distribution of median times across hospitals for a given etiology.
